# 应用超高效液相色谱-四极杆-飞行时间质谱法建立谷物中18种真菌毒素非靶向筛查数据库及确证方法

**DOI:** 10.3724/SP.J.1123.2022.05015

**Published:** 2023-01-08

**Authors:** Luxing ZHANG, Zhaohui HUANG, Shuqing LUO, Lin CAO, Ying XIE, Jiang QIAN

**Affiliations:** 1.浙江药科职业大学, 浙江 宁波 315100; 1. Zhejiang Pharmaceutical University, Ningbo 315100, China; 2.宁波市药品检验所, 浙江 宁波 315048; 2. Ningbo Institute for Drug Control, Ningbo 315048, China; 3.宁波鄞州粮食收储有限公司, 浙江 宁波 315100; 3. Ningbo Yinzhou Grain Collection and Storage Co., Ltd., Ningbo 315100, China

**Keywords:** 超高效液相色谱-四极杆-飞行时间质谱, 真菌毒素, 稻谷, 小麦, 数据库, 筛查与确证, ultra performance liquid chromatography-quadrupole-time of flight mass spectrometry (UPLC-Q-TOF/MS), mycotoxins, rice, wheat, database, screening and confirmation

## Abstract

建立了基于超高效液相色谱-四极杆-飞行时间质谱(UPLC-Q-TOF/MS)的18种真菌毒素非靶向筛查方法。真菌毒素标准物质用HSS T3色谱柱进行色谱分离后在UPLC-Q-TOF/MS MS^E^模式下分别用正、负离子模式采集,获取MS和MS/MS的信息,记录对应保留时间、加合物离子、碎片离子精确质量数等信息,设置保留时间偏移为0.3 min,加合物离子和碎片离子的精确质量匹配容差为5×10^-6^,在UNIFI中建立18种真菌毒素的数据库。在稻谷、小麦基质中,以筛查检出限(SDL)作为主要参数对筛查方法进行了验证。18种真菌毒素分为有最大限量和无最大限量两种类型,结果有最大限量的真菌毒素均能在其限量水平被准确筛查,无最大限量的真菌毒素其SDL的范围为2~800 μg/kg。基质效应考察表明,稻谷中有14种真菌毒素有中等基质效应,小麦中有11种真菌毒素有中等基质效应。样品经乙腈提取后用QuEChERS萃取盐包和HLB净化柱净化,用建立的方法对25批稻谷、小麦进行筛查,结果2批稻谷中检出4种真菌毒素,2批小麦中检出2种真菌毒素。该方法能准确筛查SDL水平以上的真菌毒素,具有高通量、简便、快捷、准确等特点,可实现无标准品情况下对稻谷、小麦中多种真菌毒素的定性筛查。

## Abstract (en)

A mass spectral library of 18 mycotoxins was developed based on ultra performance liquid chromatography-quadrupole-time of flight mass spectrometry (UPLC-Q-TOF/MS), which was used to establish a non-targeted screening method for mycotoxins in rice and wheat matrices. Eighteen mycotoxin standards were separated on an HSS T3 column, and data were collected for both positive and negative ionization under the MS^E^ mode of the UPLC-Q-TOF/MS. Details including formulas, retention times, theoretical exact masses, measured exact masses of the adduct and fragment ions, and ion abundance ratios were recorded to establish the mass spectral library of the 18 mycotoxins in UNIFI Software. Analyte detection was based on a retention time deviation of 0.3 min, and the exact mass deviation of the adduct ions and fragment ions was set to 5×10^-6^. The screening detection limit (SDL) was used as the main threshold for verifying the screening method. In the validation process, 18 mycotoxins were classified into two types: with maximum levels (MLs) and without MLs. The results showed that the mycotoxins with MLs could be accurately screened at their limited level, and the mycotoxins without MLs had a range of SDL concentration from 2 to 800 μg/kg. The matrix effect results showed that 14 mycotoxins in rice and 11 in wheat had moderate matrix effects. Finally, 25 batches of rice and wheat were purified using QuEChERS and HLB columns after acetonitrile extraction and screening were performed by employing the established method. The results revealed that aflatoxin G_1_ (AFG_1_), aflatoxin G_2_ (AFG_2_), fumonisins B_1_ (FB_1_), and sterigmatocystin (ST) were detected in one batch of rice, FB_1_ and ST were detected in another batch of rice, FB_1_ and ochratoxin A (OTA) were detected in two batches of wheat, and no other mycotoxins were detected. This method is characterized by high throughput, simplicity, rapidity, accuracy, and can be applied to accurately screen mycotoxins with concentrations higher than the SDLs and qualitatively screen various mycotoxins in rice and wheat without standards.

真菌毒素(mycotoxins)是一类由产毒真菌在特定的生长环境下产生的有毒次级代谢产物,其对谷物作物的污染严重威胁了农产品的质量与安全^[1]^。部分真菌毒素具有强毒性等特点,有些毒素会在饲用动物体内发生结构转化,以结构类似物存在于食品中,危害人类健康^[2]^。

目前发现的数百种真菌毒素中,约12种真菌毒素被认为具有严重的健康危害^[3]^,国际癌症研究机构(IARC)已将黄曲霉毒素列为Ⅰ类致癌物,将赭曲霉毒素等列为Ⅱ类致癌物^[2]^,食品安全组织及多个国家均制定了比较严格的最大残留限量标准和检测方法。但是法规覆盖的真菌毒素种类和数量有限,评估各类真菌毒素在食品的生产、加工及流通等环节的暴露风险迫在眉睫。

随着检测技术的不断更新,目前液相色谱-串联质谱(LC-MS/MS)被认为是残留检测首选的方法^[4⇓⇓⇓⇓-9]^。虽然LC-MS/MS具有较高的灵敏度和选择性,但在处理的物质种类繁多时,方法的设置繁琐且费时^[10]^;并且为了准确定性和定量,必须获得各种高浓度的真菌毒素对照品及对应的同位素内标^[11⇓-13]^,有些标准品如黄曲霉毒素类(黄曲霉素B_1_等)因纯度高、毒性大,不免会对实验者及实验环境造成一定的危害;而且由于靠预先设定的对照品来进行定性,无法获取对照品以外的真菌毒素残留信息。

随着质谱技术的发展,近年来现代液相色谱-质谱联用仪的性能改进以及有助于提升分析效率的软件工具的开发促进了残留检测技术的发展。高分辨质谱(HRMS)能够在全扫描模式下采集选定质量范围内的全部信息^[14,15]^,因此能分析几乎无限数量的污染物。它们还支持通过回顾性数据分析来查找在测定时未予考虑的污染物^[16]^。并且具有高分辨率、高灵敏性等数据采集特性^[1]^, HRMS也可以对没有参考标准的化合物进行定性筛查分析^[17]^。

本文建立了包含18种真菌毒素的UPLC-Q-TOF/MS高分辨质谱数据库,以稻谷和小麦作为研究对象,通过QuEChERS萃取盐包结合HLB净化柱的前处理方法进行筛查研究,确定每种真菌毒素在稻谷、小麦中的筛查检出限(SDL),最后将该方法应用于部分市售稻谷及小麦的真菌毒素筛查。本方法可实现无标准品情况下对真菌毒素的定性筛查,具有高通量、简便、快捷、准确等特点。

## 1 实验部分

### 1.1 仪器与试药

SYNAPT G2 MS四极杆-飞行时间高分辨质谱仪、ACQUITY UPLC超高效液相色谱仪、MassLynx V4.2工作站、UNIFI科学信息学系统(美国Waters公司); Milli-Q超纯水纯化系统(美国Millipore公司); SB 25-12 DTN超声波清洗机(宁波新芝生物科技股份有限公司)。

真菌毒素标准品溶液和标准品见表1, QuEChERS萃取盐包购自美国安捷伦公司,Oasis Prime HLB净化柱购自美国Waters公司,实验用水为Milli-Q超纯水。甲醇、乙腈(质谱纯)购自德国Merck公司,乙酸、甲酸、乙酸铵(质谱纯)购自阿拉丁试剂公司。

**表1:** 18种真菌毒素标准品和混合标准溶液

Compound	Abbreviation	Conc. of standard solution / (μg/mL)	Purity of standard substance/%	Conc. in mixed standard solution /(μg/L)	Source
Aflatoxin B_1_ (黄曲霉素B_1_)	AFB_1_	10	-	5.0	Welch
Aflatoxin B_2_ (黄曲霉素B_2_)	AFB_2_	10	-	5.0	
Aflatoxin G_1_ (黄曲霉素G_1_)	AFG_1_	10	-	5.0	
Aflatoxin G_2_ (黄曲霉素G_2_)	AFG_2_	10	-	5.0	
Ochratoxin A (赭曲霉素)	OTA	50.4	-	15.1	Sigma
Sterigmatocystin (杂色曲霉毒素)	ST	50.1	-	10.0	Romer
HT-2 toxin (HT-2毒素)	HT-2	100.4	-	50.2	
T-2 toxin (T-2)	T-2	100.4	-	10.0	Alta
Fumonisins B_1_ (伏马毒素B_1_)	FB_1_	100.3	-	100.3	
Fumonisins B_2_ (伏马毒素B_2_)	FB_2_	99.9	-	99.9	
Deoxynivalenol 3-glucoside	DON-3G	10.0	-	2000.0	
(脱氧雪腐镰刀菌烯醇-3-葡萄糖苷)					
Acetyl-deoxynivalenol (15-乙酰基脱氧雪腐镰刀菌烯醇)	15-ACDON	99.9	-	799.2	
Patulin (展青霉素)	PAT	100.0	-	1000.0	
Deoxynivalenol (脱氧雪腐镰刀菌烯醇)	DON	-	99.62	5000.0	Fermentek
Nivalenol (雪腐镰刀菌烯醇)	NIV	-	100	4000.0	
Zearalenone (玉米赤霉烯酮)	ZEN	-	99.24	300.0	
Citrinin (桔青霉素)	CIT	-	100	100.0	

Conc.: mass concentration.

### 1.2 色谱-质谱分析条件

#### 1.2.1 UPLC条件

色谱柱为Waters HSS T3 C18柱(100 mm×2.1 mm, 1.8 μm),柱温40 ℃,流动相A为含1%(体积分数)乙酸和5 mmol/L乙酸铵的水溶液,流动相B为甲醇,流速:0.3 mL/min。梯度洗脱程序:0~2.0 min, 10%B; 2.0~3.0 min, 10%B~20%B; 3.0~7.0 min, 20%B~24%B; 7.0~10.5 min, 24%B~30%B; 10.5~13.5 min, 30%B~60%B; 13.5~15.0 min, 60%B~70%B; 15.0~18.0 min, 70%B~75%B; 18.0~18.1 min, 75%B~95%B; 18.1~21.9 min, 95%B; 21.9~22.0 min, 95%B~10%B。样品进样量为3 μL。

#### 1.2.2 Q-TOF/MS条件

ESI正离子模式:毛细管电压3.00 kV,锥孔电压40 V,离子源温度110 ℃,脱溶剂气温度450 ℃,锥孔气流速50 L/h,脱溶剂气流速800 L/h。ESI负离子模式:毛细管电压3.10 kV,锥孔电压40 V,离子源温度110 ℃,脱溶剂气温度450 ℃,锥孔气流速50 L/h,脱溶剂气流速800 L/h。LockSpray溶液:亮氨酸脑啡肽(正离子*m/z* 556.2771,负离子*m/z* 554.2615);采集模式:MS^E^模式,质量扫描范围:*m/z* 50~1200;扫描时间0.25 s;低碰撞能量(LE)关闭,高碰撞能量(HE)为3~40 eV。数据采集由MassLynx V4.2工作站完成。

### 1.3 数据库的构建

#### 1.3.1 混合标准溶液的制备

按表1所示质量浓度配制混合标准溶液。

#### 1.3.2 数据库信息采集

在www.chemspider.com网站上检索真菌毒素,下载对应的.mol的文件,将列有各个真菌毒素化合物名称的excel表格和其结构式的.mol文件导入UNIFI软件,UNIFI会根据导入的结构式自动生成一份包含分子式、质量数的初步数据库。

在上述色谱-质谱条件下,UPLC-Q-TOF/MS用MS^E^模式对表1所示浓度的18种真菌毒素混合标准溶液进行采集,获得化合物的保留时间、加合物精确质量数等信息。在MS/MS模式下,得到不同碰撞能量产生的主要碎片离子的精确质量数。最后将两次数据采集的保留时间、加合物精确质量数和碎片离子的精确质量数录入数据库。

### 1.4 样品前处理

准确称取5.0 g样品于50 mL聚丙烯具塞离心管中,加入10 mL 10%(v/v)甲酸水溶液,涡旋混匀,再加入10 mL乙腈,振荡提取25 min,加入QuEChERS萃取盐包(含4 g硫酸镁、1 g氯化钠、1 g柠檬酸钠、0.5 g柠檬酸二钠盐),振荡5 min,以4000 r/min的转速离心5 min,取上清液2.5 mL加至Oasis Prime HLB小柱上,用柱塞杆稍加施压,滤液弃去0.5 mL左右,取续滤液,用0.22 μm有机滤膜过滤,进样分析。

### 1.5 UNIFI筛查条件

选取加合物(包括[M+H]^+^、[M+NH_4_]^+^、[M-H]^-^和[M+CH_3_COO]^-^)作为目标质量,低能量通道质谱报告强度阈值100,高能量通道质谱报告强度阈值20;绝对保留时间漂移0.3 min;测得离子质量数与数据库中的离子理论质量数匹配容差5×10^-6^。

## 2 结果与讨论

### 2.1 真菌毒素名单确定及其监管限量

根据中华人民共和国粮食行业标准LS/T 6133-2018《粮油检验 主要谷物中16种真菌毒素的测定 液相色谱-串联质谱法》^[18]^中发布的主要谷物中16种真菌毒素的名单,结合欧盟关于食品污染物最大限量的法规EC No. 1881/2006^[19]^,并考虑到数据库未来的应用范围,增加了现行检测标准监管较少的桔青霉素和展青霉素^[2]^,确定了构建数据库的18种真菌毒素名单,见表1。

真菌毒素有多种监管限量,具体取决于特定真菌毒素、被分析基质、监管机构和目标消费者。参考中华人民共和国国家标准GB 2761-2017《食品安全国家标准 食品中真菌毒素限量》^[20]^和EC No. 1881/2006^[19]^,在稻谷、小麦基质中被监管的真菌毒素有7种,被监管的最大限量(maximum levels,MLs)具体数据见表2。

**表2:** 本研究中涉及的真菌毒素的最大限量

Mycotoxin	GB 2761-2017		EC No. 1881/2006	
	Rice	Wheat	Rice	Wheat
AFG_1_	none	none	total: 4.0	total: 4.0
AFG_2_	none	none		
AFB_1_	10	5.0		
AFB_2_	none	none		
OTA	5.0	5.0	3.0	3.0
DON	none	1000	1250	1750
ZEN	none	60	100	100

### 2.2 数据采集条件的优化

将18种真菌毒素混合标准溶液完成数据采集后获得的相关数据信息录入UNIFI数据库(见表3),总离子流图见图1。

**表3:** 18种真菌毒素精确质量数据库

No.	Compound	Molecular formula	CAS No.	Ion mode	Theoretical precursor ion (m/z)	Experimental precursor ion (m/z)	Product ions (m/z)	t_R_/ min	m/z error/ 10^-6^
1	AFG_1_	C_17_H_12_O_7_	1165-39-5	[M+H]^+^	329.06558	329.0655	311.0525, 43.0659, 283.0544	13.91	-0.24
2	AFG_2_	C_17_H_14_O_7_	7241-98-7	[M+H]^+^	331.08123	331.0801	313.0673, 285.0701, 245.0842	13.55	-3.41
3	AFB_1_	C_17_H_12_O_6_	1162-65-8	[M+H]^+^	313.07066	313.0699	285.0716, 270.0477, 242.0516	14.55	-2.43
4	AFB_2_	C_17_H_14_O_6_	7220-81-7	[M+H]^+^	315.08631	315.0851	287.0900, 259.0551, 241.0777	14.27	-3.84
5	ST	C_18_H_12_O_6_	163391-76-2	[M+H]^+^	325.07066	325.0706	310.0475, 281.0443	17.52	-0.18
6	OTA	C_20_H_18_ClNO_6_	303-47-9	[M+H]^+^	404.08954	404.0883	358.0783, 239.0052, 166.1944	16.71	-3.07
7	T-2	C_24_H_34_O_9_	21259-20-1	[M+NH_4_]^+^	484.25411	484.2565	245.1135, 305.1354	16.22	4.94
8	FB_1_	C_34_H_59_NO_15_	116355-83-0	[M+H]^+^	722.39575	722.3990	334.3086, 352.3222	15.79	4.50
9	FB_2_	C_34_H_59_NO_14_	116355-84-1	[M+H]^+^	706.40083	706.4034	336.3198, 336.3263, 318.3171	17.52	3.64
10	CIT	C_13_H_14_O_5_	518-75-2	[M+H]^+^	251.09140	251.0922	205.0828, 191.0692	14.55	3.19
11	15-ACDON	C_17_H_22_O_7_	88337-96-6	[M+NH_4_]^+^	356.17043	356.1701	321.1331, 279.0944	12.05	-0.92
12	DON	C_15_H_20_O_6_	51481-10-8	[M+CH_3_COO]^-^	355.13985	355.1409	295.1158, 265.1033, 247.0968	4.95	2.96
13	NIV	C_15_H_20_O_7_	23282-20-4	[M+CH_3_COO]^-^	371.13475	371.1353	311.1050, 281.0974	3.36	1.48
14	ZEN	C_18_H_22_O_5_	17924-92-4	[M-H]^-^	317.13945	317.1382	273.1464, 175.0364, 149.0561	17.08	-3.94
15	DON-3G	C_21_H_30_O_11_	131180-21-7	[M+CH_3_COO]^-^	517.19265	517.1905	457.1694, 427.1625	5.10	-4.16
16	HT-2	C_22_H_32_O_8_	26934-87-2	[M+CH_3_COO]^-^	483.22355	483.2249	59.0111, 141.0143	15.50	2.79
17	3-ACDON	C_17_H_22_O_7_	50722-38-8	[M+CH_3_COO]^-^	397.15040	397.1516	337.1325, 307.1198, 173.0569	11.94	3.02
18	PAT	C_7_H_6_O_4_	149-29-1	[M-H]^-^	153.01932	153.0189	109.0329	2.87	-2.94

**图1:**
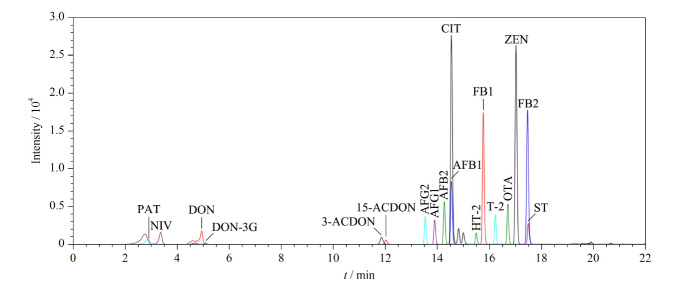
18种真菌毒素的总离子流色谱图

在数据库的建立中,为了获得精确的加合物离子质量数和主要碎片离子质量数,我们在MassLynx中选择MS/MS模式,根据不同真菌毒素的结构特性,为每一个真菌毒素设立一组碰撞电压,记录加合物离子的精确质量数和主要碎片离子的精确质量数。如AFG_1_,考察了在碰撞能量(CE)分别为10、20、25 eV的情况下,加合物离子和碎片离子的分布和响应,选择20 eV为其最佳碰撞能量,并记录加合物离子的精确质量数为329.0655,主要碎片离子的精确质量数为311.0525、243.0659和283.0544,且该碎片离子的响应为加合物离子响应的10%以上。

确定特定碰撞能量下的加合物离子和子离子之后,用于样品筛查的色谱-质谱图需要在MassLynx中选择分辨率模式下的MS^E^模式采集。MS^E^的特点是能够获得包含所有成分的MS及MS/MS数据信息^[21]^。因此我们在MS^E^模式下同时开启低能量和高能量采集通道,高能量通道中设置了梯度碰撞能量,为3~40 eV,涵盖了在MS/MS模式下每一个真菌毒素的碰撞能量。

其中11种真菌毒素用正离子模式采集,7种真菌毒素用负离子模式采集。如果均以正离子或者负离子的模式采集会导致部分真菌毒素的响应达不到最佳状态。另外引入碎片离子这一维度信息,减少了假阳性的产生。

### 2.3 筛查方法的设置

将包含有色谱、质谱信息的原始数据导入UNIFI科学信息学系统,基本筛查流程见图2。

**图2:**
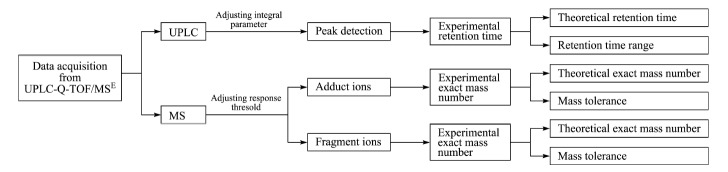
数据库的筛查流程图

由于真菌毒素类筛查方法尚未给出明确指南,我们借鉴欧盟SANTE/11813/2017指南《食品和饲料中农药残留分析的方法验证和质量控制程序》^[22]^中关于高分辨质谱筛查结果的鉴定与确证方法,必须满足以下4个条件的前三条:第一,目标峰信噪比≥3;第二,至少两个测得的质量数(其中一个是碎片离子)与理论质量数质量偏差不超过5×10^-6^;第三,在基质匹配条件下,加合物离子和碎片离子的提取色谱图保留时间重合(±0.1 min);第四,试样与标准溶液中离子相对丰度的偏差最好不超过30%。但指南中也提到,第四个指标匹配离子相对丰度的偏差的要求不那么严格,可能随实验方法和仪器的稳定性不同而发生偏移,需具体情况具体分析,但应作为指示值,超过30%应进一步调查并判断。

使用UNIFI数据库进行自动筛查时,所设置的筛查条件直接影响筛查结果。筛查结果需要同时满足保留时间和精确质量数两个条件。通过积分参数的设置进行色谱峰识别,并将“已识别”的目标组分与数据库中指定的保留时间进行匹配。色谱系统虽然有较好的稳定性,但由于基质等原因会造成样品的保留时间与数据库中用标准品录入的保留时间之间会有一定的偏差,因此我们将保留时间窗口设置为0.3 min。在质谱方面,需要通过严格的质量数范围来筛选目标,同时考虑仪器分辨率的稳定性和筛查的准确性,加合物离子和碎片离子的质量数匹配容差设为5×10^-6^。此外,在进行加合物精确质量数匹配时,筛选候选项中的所有同位素,识别同位素的匹配情况,以避免产生假阴性结果。用真菌毒素混合标准溶液验证设置的筛查方法,结果显示能将目标物全部准确标识,没有发生错配的现象。

以3-ACDON为例,通过与数据库比对预期保留时间(11.94 min)和预期的精确质量数(337.1325、307.1198、173.0569),就能在混合物中筛出3-ACDON并做标识。在色谱通道中,找到了加合物离子(*m/z* 397.1519)和特征碎片离子(*m/z* 337.1312、307.1210)对应的色谱峰,该色谱峰的保留时间如图3所示,加合物离子的保留时间(11.84 min)和碎片离子的保留时间(11.84 min)完全一致。UNIFI软件给出了低、高两个碰撞能量通道的质谱图,3-ACDON在高碰撞能量通道中,2个主要碎片离子(*m/z* 337.13125、307.12097)与数据库中的碎片离子的精确质量数偏差分别为3.7×10^-6^和3.8×10^-6^。符合SANTE/11813/2017指南中的要求“至少两个测得的质量数(其中一个是碎片离子)与理论质量数质量偏差不超过5×10^-6^”。此外软件给出了碎片离子(*m/z* 307.12097、173.06271)可能的结构,如图4所示。

**图3:**
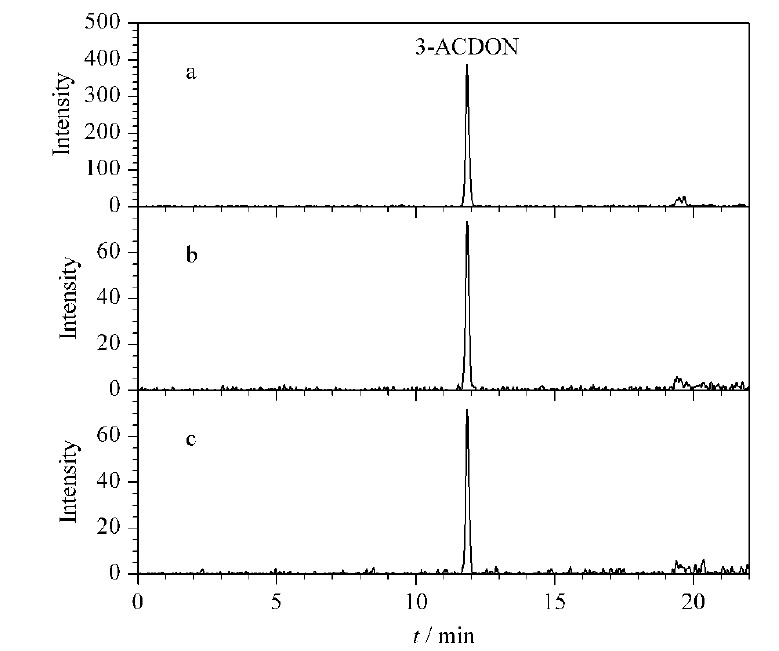
3-ACDON的筛查分析色谱图

**图4:**
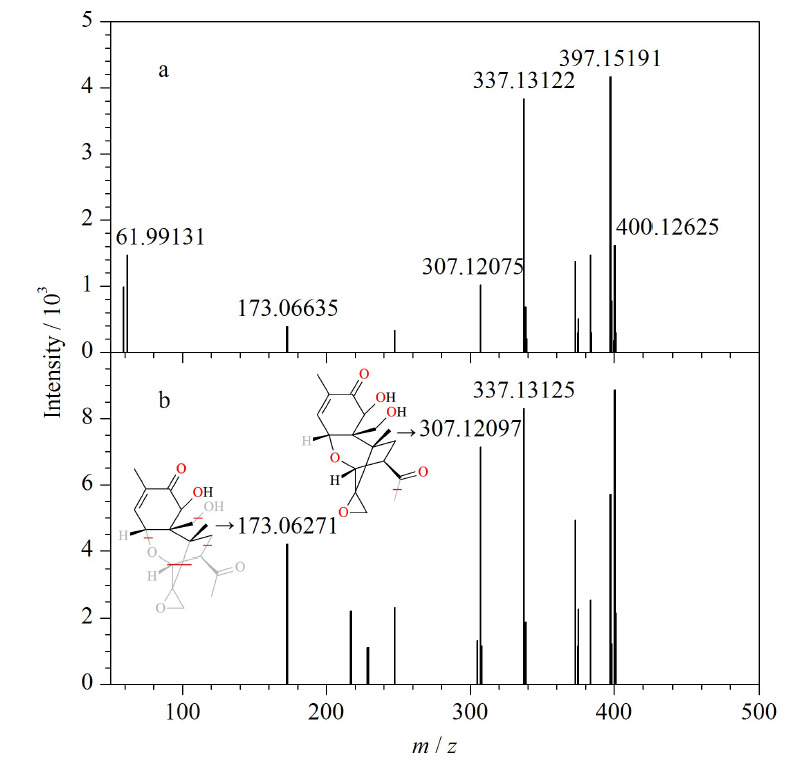
3-ACDON的低能量和高能量通道筛查质谱图

### 2.4 筛查方法学验证

由于本方法侧重于定性筛查,因此重点验证筛查方法的准确性与稳定性。本文参考SANTE/11813/2017指南,以筛查检出限作为主要验证参数^[22]^。在稻谷、小麦基质中,18种真菌毒素中有7种真菌毒素规定了MLs, 11种尚未规定,按照SDL的定义“目标物在至少95%的样本中被检测到的最低浓度(不一定符合MS鉴定标准)”^[22]^,我们把SDL的水平分成两类,一类是已有MLs的真菌毒素,由于这类真菌毒素其限量浓度高低不一,我们无法用绝对的数值去评估,因此这类真菌毒素以0.5倍、1倍、2倍、4倍MLs作为筛查浓度,做筛查方法学验证;另一类是没有规定MLs的真菌毒素,则以本方法中高分辨质谱能做到的至少95%的样品中可以检测到的最低浓度作为SDL。其中MLs是综合参考GB 2761-2017与EC No. 1881/2006,若针对同一个真菌毒素有不同的限量规定,则选取较低的MLs作为本文的依据。

选取25份稻谷、小麦,精密量取混合标准溶液0.5、1.0、2.0、4.0 mL添加至5 g样品中,经样品前处理后进行筛查检测。通过对检测结果的分析,确定小麦和稻谷中18种真菌毒素的SDL,结果见表4和表5。

**表4:** 18种真菌毒素在稻谷中的加标水平、检出个数及确定的筛查检出限

No.	Compound	Level 1			Level 2			Level 3			Level 4				SDL/ (μg/kg)
		Content/ (μg/kg)	Number detected^*^		Content/ (μg/kg)	Number detected^*^		Content/ (μg/kg)	Number detected^*^		Content/ (μg/kg)		Number detected^*^		
1	AFG_1_	0.5	13		1.0	24		2.0	25		4.0	25		1.0	
2	AFG_2_	0.5	18		1.0	24		2.0	25		4.0	25		1.0	
3	AFB_1_	0.5	19		1.0	24		2.0	24		4.0	25		1.0	
4	AFB_2_	0.5	18		1.0	24		2.0	24		4.0	25		1.0	
5	OTA	1.5	19		3.0	24		6.0	24		12.0	25		3.0	
6	DON	500.0	19		1000	24		2000	25		4000	25		1000	
7	ZEN	30	25		60	25		120	25		240	-		<30	
8	ST	1.0	20		2.0	24		4.0	24		8.0	-		2.0	
9	T-2	1.0	-		2.0	16		4.0	21		8.0	24		8.0	
10	CIT	10.0	-		20.0	15		40.0	18		80.0	24		80.0	
11	FB_1_	10.0	16		20.0	24		40.0	24		-	-		20.0	
12	FB_2_	10.0	15		20.0	24		40.0	24		-	-		20.0	
13	NIV	400.0	18		800.0	24		1600	25		-	-		800.0	
14	DON-3G	200.0	-		400.0	21		800.0	24		1600	24		800.0	
15	HT-2	5.0	-		10.0	18		20.0	22		40.0	25		40.0	
16	3-ACDON	40.0	21		80.0	24		160	25		320	-		80.0	
17	15-ACDON	79.9	-		159.8	17		319.6	19		639.2	24		639.2	
18	PAT	100.0	-		200.0	15		400.0	23		800.0	24		800.0	

-: There is no need for statistics. * in 25 samples.

**表5:** 18种真菌毒素在小麦中的加标水平、检出个数及确定的筛查检出限

No.	Compound	Level 1			Level 2			Level 3			Level 4				SDL/ (μg/kg)
		Content/ (μg/kg)	Number detected^*^		Content/ (μg/kg)	Number detected^*^		Content/ (μg/kg)	Number detected^*^		Content/ (μg/kg)		Number detected^*^		
1	AFG_1_	0.5	15		1.0	24		2.0	25		4.0	25		1.0	
2	AFG_2_	0.5	16		1.0	24		2.0	25		4.0	25		1.0	
3	AFB_1_	0.5	18		1.0	24		2.0	25		4.0	25		1.0	
4	AFB_2_	0.5	16		1.0	25		2.0	24		4.0	25		1.0	
5	OTA	1.5	20		3.0	24		6.0	25		12.0	25		3.0	
6	DON	500.0	20		1000	24		2000	25		4000	25		1000	
7	ZEN	30	25		60	25		120	25		240	-		<30	
8	ST	1.0	21		2.0	24		4.0	25		8.0	-		2.0	
9	T-2	1.0	-		2.0	19		4.0	22		8.0	24		8.0	
10	CIT	10.0	-		20.0	14		40.0	19		80.0	24		80.0	
11	FB_1_	10.0	16		20.0	24		40.0	25		-	-		20.0	
12	FB_2_	10.0	16		20.0	24		40.0	24		-	-		20.0	
13	NIV	400.0	18		800.0	25		1600	25		-	-		800.0	
14	DON-3G	200.0	-		400.0	22		800.0	24		1600	25		800.0	
15	HT-2	5.0	-		10.0	19		20.0	24		40.0	25		20.0	
16	3-ACDON	40.0	19		80.0	25		160	25		320	-		80.0	
17	15-ACDON	79.9	-		159.8	19		319.6	22		639.2	24		639.2	
18	PAT	100.0	-		200.0	16		400.0	21		800.0	24		800.0	

-: There is no need for statistics. * in 25 samples.

由表4和表5可知,对于已有MLs的真菌毒素,AFB_1_、AFB_2_、AFG_1_、AFG_2_、OTA、DON均可在稻谷、小麦基质中在其各自的MLs水平实现95%的样品被筛查出,其中ZEN在0.5倍MLs,即30 μg/kg的水平也能被准确筛查,则其SDL小于MLs(60 μg/kg)。对于目前没有规定MLs的真菌毒素,以ST毒素为例,在加标水平分别是1.0、2.0、4.0、8.0 μg/kg处,统计能达到筛查标准的样品数量。在稻谷基质中,在加标水平为1.0 μg/kg处能被准确筛查到的样品数为21个,我们查看未被筛查到的4个样品数据信息,发现信噪比基本在10左右,保留时间基本一致,但是加合物离子的实测精确质量数与理论精确质量数偏差大于5×10^-6^,因此未被本法识别。当其加标水平增大到2.0 μg/kg时,在稻谷基质中,能被准确筛查到的样品数为24个,符合筛查条件,也满足了色谱峰信噪比≥3,加合物离子和碎片离子提取色谱峰保留时间一致。同样的,当含量达到4.0 μg/kg时,在稻谷基质中,能被准确筛查到的样品数依然为24个。鉴于在上述3种浓度条件下的筛查结果,可以认为ST毒素在稻谷基质上的SDL为2.0 μg/kg,最高水平8.0 μg/kg的数据不必统计,在表格中以“-”表示。同理,对于T-2毒素,当2.0、4.0 μg/kg均达不到筛查要求时,对于最低水平1.0 μg/kg的数据也不做统计,在表格中以“-”表示。最终确定没有MLs的真菌毒素的SDL数据见表4和表5,最低的SDL为2.0 μg/kg,为ST毒素;最高的SDL为800.0 μg/kg,为NIV、DON-3G、PAT。

考虑到样品本身会有真菌毒素污染,在做筛查检出限验证之后,对空白样品进行了一次筛查,1份稻谷中检出AFG_1_、AFG_2_、FB_1_、ST, 1份稻谷中检出FB_1_、ST, 2份小麦中检出FB_1_、OTA(见2.6节)。由于受限于样品被污染的多样性、基质效应和仪器检测灵敏度等因素的制约,我们无法获得绝对不含18种真菌毒素的空白稻谷、小麦样品,样品本底的污染会影响被监控真菌毒素SDL的准确性。为减少制约因素带来的误差,采用的措施如下:选用25批稻谷、小麦样品,均为1年内新粮,储存条件良好,减少被真菌毒素污染的几率;对2.6节所述样品进行3倍体积进样(10 μL),结果仍与2.6节结果一致,即同样的1份稻谷中检出AFG_1_、AFG_2_、FB_1_、ST, 1份稻谷中检出FB_1_、ST,同样的2份小麦中检出FB_1_、OTA,其他真菌毒素均未检出。

最后的SDL认定以稻谷中ST毒素为例,当加标水平为2.0 μg/kg时,在统计数量过程中,25批中检出24批,则有95%的样品被筛查到,但其中包含了2批本底受污染的样品,其检出的真菌毒素是本底和混合标样两个浓度的叠加,考虑到此种情况,计算时扣掉2批本底受污染的样品。剩下的23批可认为是未被18种真菌毒素污染的样品,结果23批中检出22批,依然可达到95%。

### 2.5 基质效应

在稻谷、小麦提取净化后,可能会有一些共同提取物对目标真菌毒素的离子化效率产生影响,造成目标物的信号响应增强或抑制。针对定性筛查方法,相关指南并没有给出基质效应的评价方法,由于基质效应会增强或者抑制离子信号,可能会造成假阴性或者假阳性的可能,因此我们对定性筛查的基质效应做初步考察。

选取自身受真菌毒素污染相对较低的3批稻谷、小麦样品作为空白基质,按照1.4节进行提取净化,获得空白基质提取液。用空白基质提取液和稀释液分别配制表4中4个质量浓度的混合标准溶液,同法分析。ME=(基质匹配校准曲线的斜率-标准曲线斜率)/标准曲线斜率×100%,当|ME|<20%时,弱基质效应,可以忽略;20%≤|ME|≤50%,中等基质效应;|ME|>50%,强基质效应。如图5所示,在小麦基质中,有7种真菌毒素|ME|<20%,是弱基质效应,有11种真菌毒素20%<|ME|<50%,是中等基质效应;在稻谷基质中,有4种真菌毒素|ME|<20%,是弱基质效应,有14种真菌毒素20%<|ME|<50%,是中等基质效应。对于有MLs的真菌毒素,虽然最高的基质抑制达46%,但其在限量浓度水平,提取离子色谱图中目标物的信噪比大于3,且加合离子和其中一个碎片离子精确质量数与数据库质量偏差≤5×10^-6^,其限量浓度达到了筛查检出限的水平,可避免假阴性的可能;对于没有MLs的真菌毒素,其中15-ACDON、DON-3G、桔青霉素及展青霉素的基质抑制超过45%,表4和表5显示,它们的SDL则较其他真菌毒素高。可能的原因是本文中的净化方法以尽量满足全部目标物的提取而牺牲了各个真菌毒素的回收率,这种为了获得提取的普适性而降低针对性的操作,在非靶向筛查中较为常见^[23,24]^。

**图5:**
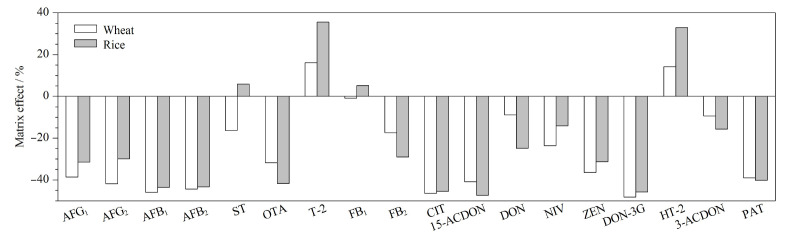
稻谷、小麦中18种真菌毒素的基质效应

### 2.6 实际样品检测

采用本文建立的方法对市售25批稻谷、小麦进行了18种真菌毒素的分析筛查验证,各筛查出2份阳性样品,1份稻谷中检出AFG_1_、AFG_2_、FB_1_、ST, 1份稻谷中检出FB_1_、ST; 2份小麦中检出FB_1_、OTA,其他真菌毒素均未检出。以一份检出ST的稻谷样品为例,将样品采集的数据与数据库中的保留时间、加合物离子和碎片离子的精确质量数进行比对,ST的提取离子色谱图和质谱图分别见图6和图7。在色谱图中,系统通过数据库标识了筛查出来的组分名字和保留时间,在高能量通道,可以观测到提取的碎片离子(*m/z* 310.0455、281.0436)对应的色谱峰,其保留时间(17.48 min、17.47 min)和ST [M+H]^+^加合物离子的保留时间(17.47 min)一致。在质谱图的低能量通道,有[M+H]^+^加合物离子峰的精确质量数325.07047,与其理论精确质量数偏差为0.6×10^-6^。在质谱图的高能量通道,有特征碎片离子*m/z* 281.04360,与理论精确质量数偏差为2.4×10^-6^,因此加合物离子与碎片离子测得精确质量数与理论精确质量数均在5×10^-6^以内。另外*m/z* 281.04360这个碎片离子相对丰度与标准溶液相对丰度的偏差均大于30%。可能影响碎片离子相对丰度偏差的因素主要包括仪器设备的稳定性、样品基质的影响及目标碎片离子较低的响应(*S/N*<15)^[16]^。指南中指出,由于精确质量测量的附加因素,对匹配离子相对丰度偏差的要求不那么严格,但应作为指示值,超过30%应进一步调查并仔细判断。因此后续可通过调整前处理的方式尝试样品浓缩来提高信号的响应,再对离子相对丰度的偏差进行评估。筛查结果表明,超过了SDL浓度的真菌毒素一定能检出,这对于可疑物质的风险控制较为有利。但同时,我们认为检出不代表一定超出MLs,对于检出的真菌毒素的浓度具体是多少,还需LC-MS/MS进行定量。

**图6:**
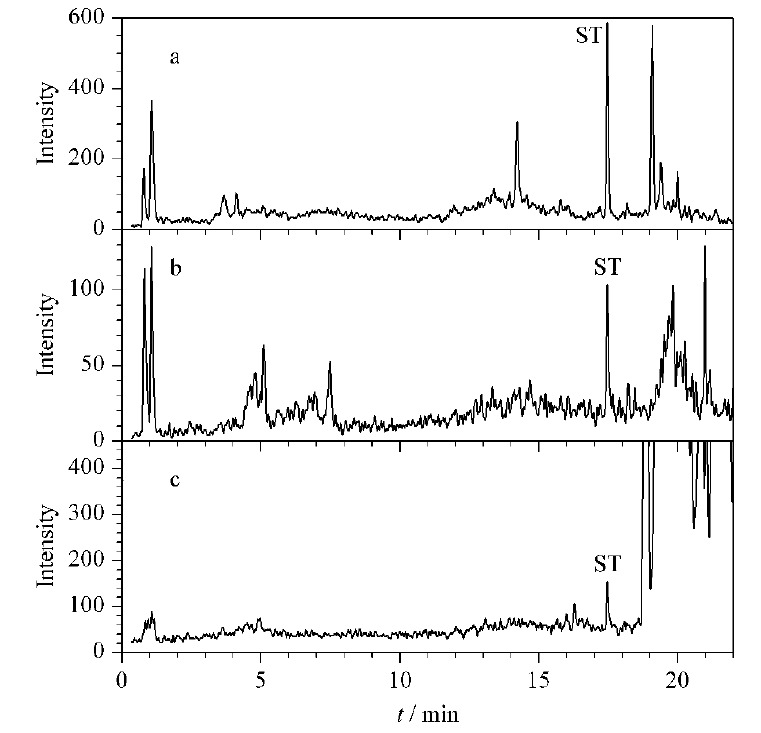
水稻中ST的筛查分析色谱图

**图7:**
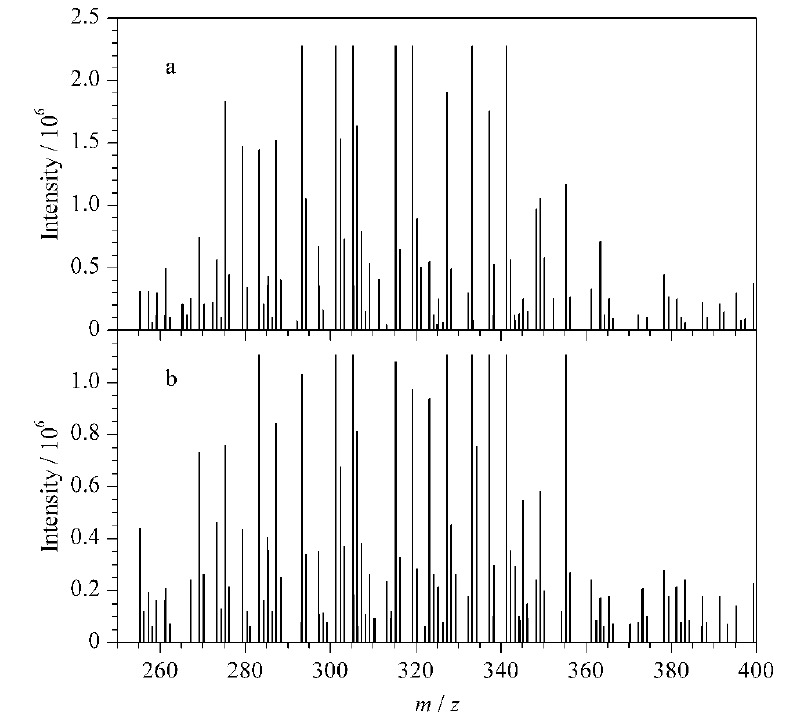
水稻中ST的筛查分析质谱图

## 3 结论

本研究采用超高效液相色谱-四极杆-飞行时间高分辨质谱仪建立了真菌毒素非靶向筛查数据库,并针对稻谷及小麦基质进行筛查方法的开发和验证,最后将该方法应用于市售稻谷及小麦中真菌毒素的筛查。可实现无标准品情况下对真菌毒素的定性筛查。该法具有高通量、简便、快捷、准确等特点,适用于稻谷、小麦中多种真菌毒素的定性检测,同时也降低了检测人员被多品种、高浓度真菌毒素标准品危害的几率,为粮谷中的真菌毒素筛查检测提供新思路与新方法,也为监管提供技术支持。
